# Uncovering Relationships between the Electronic Self-Energy and Coupled-Cluster Doubles Theory

**DOI:** 10.1021/acs.jpca.5c03750

**Published:** 2025-09-10

**Authors:** Christopher J. N. Coveney

**Affiliations:** Department of Physics, 6396 University of Oxford, Oxford OX1 3PJ, U.K.

## Abstract

We derive the coupled-cluster doubles (CCD) amplitude equations by introduction of the particle-hole-time decoupled electronic self-energy. The resulting analysis leads to an expression for the ground-state correlation energy that is exactly of the form obtained in coupled-cluster doubles theory. We demonstrate the relationship to the ionization potential/electron affinity equation-of-motion coupled-cluster doubles (IP/EA-EOM-CCD) eigenvalue problem by coupling the reverse-time self-energy contributions while maintaining particle-hole separability. The formal relationships established are demonstrated by exact solution of the Hubbard dimer.

## Graphical abstract

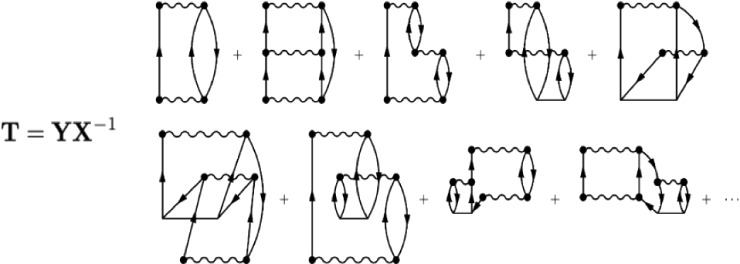

## 1 Introduction

Green’s function methods simultaneously encode excited and ground state many-body correlation. This is reflected by the fact that exact ground state properties as well as the single-particle charged excitation spectrum can be obtained from the same single-particle Green’s function.^1–3^ Recently there has been significant interest in identifying and leveraging connections between coupled-cluster and Green’s function theory.^4–16^ In this work, we uncover the connection between the electronic self-energy and the coupled-cluster doubles amplitude equations by showing how, under certain approximations, the “upfolded” quasiparticle equation can be recast to give the CCD amplitude equations. In the following, indices *i, j, k*, ... denote occupied (valence band) spin–orbitals, *a, b, c*, ... virtuals (conduction band) and *p, q, r*, ... general spin–orbitals. We employ a real canonical spin–orbital basis throughout this work.

## 2 Green’s Function Theory and the Algebraic Diagrammatic Construction Method

The central quantity in the perturbation theory of the single-particle Green’s function is the self-energy. Through the Dyson equation, an approximation for the self-energy defines the corresponding approximation for the single-particle Green’s function. The electronic self-energy has a spectral representation that is a consequence of the analytic structure of the single-particle Green’s function and is given by^15–20^

1 $$\begin{array}{ll} \Sigma_{pq}\left(\omega \right) & =\Sigma_{pq}^{\infty }+\Sigma_{pq}^{F}\left(\omega \right)+\Sigma_{pq}^{B}\left(\omega \right)\\ & =\Sigma_{pq}^{\infty }+\underset{JJ^{\prime }}{\sum }U_{pJ}^{†}{[(\omega +i\eta )l-(K^{>}+C^{>})]}_{JJ^{\prime }}^{-1}U_{J^{\prime }q}\\ & +\underset{AA^{\prime }}{\sum }V_{pA}{[(\omega -i\eta )l-(K^{<}+C^{<})]}_{AA^{\prime }}^{-1}V_{A^{\prime }q}^{†} \end{array}$$

$\Sigma_{pq}^{\infty }$ is the static, frequency-independent contribution to the electronic self-energy and $\Sigma_{pq}^{F/B}\left(\omega \right)$ are the dynamical forward-/backward-time self-energy contributions, respectively. From the spectral representation of the self-energy, it can be shown that the *eigenvalue*-self-consistent frequency-dependent quasiparticle equation is equivalent to diagonalization of the “upfolded” frequency-independent Dyson supermatrix^8,15,16,19–22^

2 $$D=\left(\begin{array}{lll} f_{pq}+\Sigma_{pq}^{\infty } & U_{pJ^{\prime }}^{†} & V_{pA^{\prime }}\\ U_{Jq} & \left(K_{JJ^{\prime }}^{>}+C_{JJ^{\prime }}^{>}\right) & 0\\ V_{Aq}^{†} & 0 & \left(K_{AA^{\prime }}^{<}+C_{AA^{\prime }}^{<}\right) \end{array}\right)$$

The composite indices *JJ*′/*AA*′ label forward-time/backward-time Intermediate State Configurations (ISCs) and outline the character of the different multiparticle-hole configurations that connect the initial and final Green’s function spin–orbital indices as a result of interaction and particle propagation in the system. ISCs are excited state configurations containing (*N* ± 1)-electrons that can be related to specific electronic configurations resulting from excitation with respect to a general reference state.^15–17,19,23,24^ The matrices $\left(K_{JJ^{\prime }}^{>}+C_{JJ^{\prime }}^{>}\right)$ and $\left(K_{AA^{\prime }}^{<}+C_{AA^{\prime }}^{<}\right)$ represent the interactions between the different ISCs. The quantities *U*_*qJ*_ and *V*_*pA*_ represent the coupling matrices that link initial and final single-particle Green’s function indices to the ISCs. The upper left block of **D** is defined over the complete set of occupied and virtual spin–orbitals, where *f*_*pq*_ = ϵ_*p*_δ_*pq*_ is the Fock operator. The coupling and interaction matrices contain coupling to all possible ISCs. The zero entries of the Dyson supermatrix are present as the forward-and backward-time self-energy contributions are coupled only through the initial and final single-particle Green’s function indices. Diagonalization of the Dyson supermatrix yields the complete set of ionization potentials and electron affinities:

$$D\;\left(\begin{array}{l} A_{p}^{\nu }\\ W_{J}^{+,\nu }\\ W_{A}^{-,\nu } \end{array}\right)=\;\left(\begin{array}{l} A_{p}^{\nu }\\ W_{J}^{+,\nu }\\ W_{A}^{-,\nu } \end{array}\right)\varepsilon_{\nu }$$ 3

where ε_ν_ are the exact poles of the Green’s function. The inverse of the norm of the eigenvectors yields the quasiparticle renormalization factor as^16^

4 $$\begin{array}{ll} Z_{\nu } & ={\left(\underset{p}{\sum }A_{p}^{\nu *}A_{p}^{\nu }+\underset{J}{\sum }W_{J}^{+,\nu *}W_{J}^{+,\nu }+\underset{A}{\sum }W_{A}^{-,\nu *}W_{A}^{-,\nu }\right)}^{-1}\\ & ={\left(1-\frac{\partial \Sigma_{\nu \nu }\left(\omega \right)}{\partial \omega }{|}_{\varepsilon_{\nu }}\right)}^{-1} \end{array}$$

where we have implicitly enforced the normalization condition: $\underset{p}{\sum }A_{p}^{\nu *}A_{p}^{\nu }=1$ . The sum rule is given by $\underset{\nu }{\sum }Z_{\nu }=N_{e}$ , where *N*_*e*_ is the number of electrons.^25,26^

The third-order Algebraic Diagrammatic Construction method, ADC(3), provides an infinite-order partial summation of a third-order diagrammatic perturbation expansion of the electronic self-energy.^17,19^ It combines diagonalization of the Dyson supermatrix with perturbation expansions of the coupling and interaction matrix elements of the self-energy. For a complete account of the ADC­(*n*) method, the reader is referred to refs 17, 19 , and^20^. In this work we simply state the results. The forward-time coupling matrices are defined as

5a $$\begin{array}{ll} U_{p,iab}^{†} & =\left\langle pi\left|\right|ab\right\rangle +\frac{1}{2}\underset{kl}{\sum }\left\langle pi\left|\right|kl\right\rangle (t_{kl}^{ab}{)}_{\mathrm{MP}2}\\ & -\underset{kc}{\sum }\left\langle pc\left|\right|bk\right\rangle (t_{ki}^{ac}{)}_{\mathrm{MP}2}+\underset{kc}{\sum }\left\langle pc\left|\right|ak\right\rangle (t_{ki}^{bc}{)}_{\mathrm{MP}2} \end{array}$$

with the backward-time contributions as

5b $$\begin{array}{ll} V_{p,ija} & =\left\langle pa\left|\right|ij\right\rangle +\frac{1}{2}\underset{bc}{\sum }\left\langle pa\left|\right|bc\right\rangle (t_{ij}^{bc}{)}_{\mathrm{MP}2}\\ & -\underset{kb}{\sum }\left\langle pk\left|\right|jb\right\rangle (t_{ki}^{ba}{)}_{\mathrm{MP}2}+\underset{kb}{\sum }\left\langle pk\left|\right|ib\right\rangle (t_{kj}^{ba}{)}_{\mathrm{MP}2} \end{array}$$

Here, we introduce the MP2 CCD amplitude notation as $(t_{ij}^{ab}{)}_{\mathrm{MP}2}=-\frac{\left\langle ab\left|\right|ij\right\rangle }{\Delta_{ij}^{ab}}$ , with $\Delta_{ij}^{ab}=\epsilon_{a}+\epsilon_{b}-\epsilon_{i}-\epsilon_{j}$ and ⟨*pq*||*rs*⟩ = ⟨*pq*|*rs*⟩ – ⟨*pq*|*sr*⟩ representing the antisymmetrized two-electron repulsion integrals. The forward-time interaction matrices are given by

6a $$\begin{array}{ll} \left(K_{iab,jcd}^{>,2p1h}+C_{iab,jcd}^{>,2p1h}\right) & =\left(\epsilon_{a}+\epsilon_{b}-\epsilon_{i}\right)\left(\delta_{ac}\delta_{bd}-\delta_{ad}\delta_{bc}\right)\delta_{ij}\\ & +\left\langle ab\left|\right|cd\right\rangle \delta_{ij}+\left\langle jb\left|\right|di\right\rangle \delta_{ac}\\ & -\left\langle jb\left|\right|ci\right\rangle \delta_{ad}+\left\langle ja\left|\right|ci\right\rangle \delta_{bd}\\ & -\left\langle ja\left|\right|di\right\rangle \delta_{bc} \end{array}$$

with the backward-time interaction matrices as

6b $$\begin{array}{ll} \left(K_{ija,klb}^{<,2.h1p}+C_{ija,klb}^{<,2h1p}\right) & =\left(\epsilon_{i}+\epsilon_{j}-\epsilon_{a}\right)\delta_{ab}\left(\delta_{ik}\delta_{jl}-\delta_{il}\delta_{jk}\right)\\ & -\left\langle ij\left|\right|kl\right\rangle \delta_{ab}-\left\langle jb\left|\right|al\right\rangle \delta_{ik}\\ & +\left\langle jb\left|\right|ak\right\rangle \delta_{il}+\left\langle ib\left|\right|al\right\rangle \delta_{jk}\\ & -\left\langle ib\left|\right|ak\right\rangle \delta_{jl} \end{array}$$

Using these expressions for the coupling and interaction matrices in the Dyson supermatrix representation (eq 2) corresponds to an infinite-order summation of the electronic self-energy diagrams depicted in Figure 1. To identify the connection to coupled-cluster doubles theory, we define Σ^∞^ = 0, as the reference zeroth-order Hamiltonian is the Fock operator.

**Figure 1:**
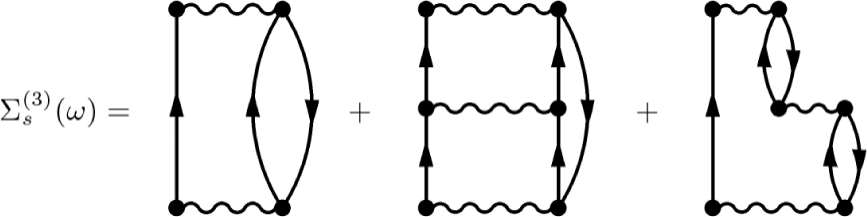
Third-order one-particle irreducible skeleton electronic self-energy diagrams. The ADC(3) Dyson supermatrix performs an infinite-order summation of these terms.

## 3 Relationship between the Electronic Self-Energy and Coupled-Cluster Doubles Theory

In the following we show that the connection between the electronic self-energy and the CCD amplitude equations can be derived by decoupling the forward and backward time self-energy components along with separating the particle-hole sectors. The decoupling of the different time directions and particle-hole sectors is commonly referred to as a non-Dyson self-energy approximation.^8,10,27,28^

The connections presented in this section are related to but distinct from the diagrammatic coupled-cluster self-energy, recently introduced in refs 15 and 16, which is defined over the complete set of occupied and virtual orbitals. As a result, the non-Dyson electronic self-energy uncovered in this work that yields the exact CCD amplitude equations cannot be derived by taking functional derivatives of a diagrammatic expansion for the CC ground-state energy.

We refer to this non-Dyson electronic self-energy approximation as the *particle-hole-time decoupled self-energy*. For the occupied states, we have

7 $${\mathrm{\Sigma ̅}}_{ij}^{F}(\omega )=\underset{\begin{array}{l} ka>b\\ lc>d \end{array}}{\sum }{\mathrm{U̅}}_{i,kab}\times {\left(\left(\omega +i\eta \right)l-\left(K^{>,2p1h}+C^{>,2p1h}\right)\right)}_{kab,lcd}^{-1}U_{lcd,j}$$

where all backward-time contributions are decoupled. This is motivated by the fact that the coupled-cluster excitation operators contain only hole to particle excitations and so are restricted to only contain forward-time propagation: $T_{2}=\sum_{\begin{array}{l} b>a\\ j>i \end{array}}t_{ij}^{ab}a_{a}^{†}a_{b}^{†}a_{j}a_{i}$ . Additionally, we also neglect all forward-time self-energy diagrams that contain internal backward-time propagation of the intermediate Green’s function lines from the final interaction vertex such that ${\mathrm{U̅}}_{i,jab}=\left\langle ij\left|\right|ab\right\rangle$ . This procedures ensures that the correct set of only forward-time ordered Feynman-Goldstone self-energy diagrams are included in the particle-hole-time decoupled self-energy. However, the form of the second coupling matrix, *U_lcd,j_* remains the same as defined in eq 5. In general, this breaks the hermiticity of the coupling elements of the electronic self-energy. This retention of specific forward-time ordered diagrams is closely related to well-known approximations for the polarization propagator. The full set of time-orderings contained in the ADC(3) supermatrix contain both forward- and backward-time bubble, exchange and ladder polarization insertions. This is also the case in the *GW*-RPA approximation whereby both forward- and backward-time bubble diagrams are present.^6,10^ The inclusion of both time-orderings of these polarization diagrams is particularly important for both electronic relaxation and correlation processes.

By particle-hole symmetry, we have the particle-hole-time decoupled self-energy of the virtual states as

8 $${\mathrm{\Sigma ̅}}_{ab}^{B}\left(\omega \right)=\underset{\begin{array}{l} i>jc\\ k>ld \end{array}}{\sum }{\mathrm{V̅}}_{a,ijc}\times {\left(\left(\omega -i\eta \right)l-\left(K^{<,2h1p}+C^{<,2h1p}\right)\right)}_{ijc,kld}^{-1}V_{kld,b}^{†}$$

where now we decouple all forward-time contributions and neglect all diagrams that contain internal forward-time propagation of the intermediate Green’s function lines from the final interaction vertex such that ${\overset{¯}{V}}_{a,ijc}=\left\langle ac\left|\right|ij\right\rangle$ . The second coupling matrix element, $V_{kld,b}^{†}$ , remains the same as defined in eq 6. Decoupling the particle-hole sectors requires: ${\mathrm{\Sigma ̅}}_{ia}={\mathrm{\Sigma ̅}}_{ai}=0$ .

Focusing on the particle-hole-time decoupled self-energy of the occupied-occupied block, eq 9, we have the upfolded supermatrix eigenvalue problem

$$\left(\begin{array}{ll} \epsilon_{i}\delta_{ij} & {\mathrm{U̅}}_{i,jab}\\ U_{kcd,i} & \left(K_{iab,jcd}^{>,2p1h}+C_{iab,jcd}^{>,2p1h}\right) \end{array}\right)\left(\begin{array}{l} X_{i}^{\nu }\\ Y_{iab}^{\nu } \end{array}\right)=\left(\begin{array}{l} X_{i}^{\nu }\\ Y_{iab}^{\nu } \end{array}\right)\varepsilon_{\nu }$$ 9

Now, we introduce the doubles amplitudes through the follow identity: $t_{ij}^{ab}=\underset{\nu }{\sum }Y_{jab}^{\nu }(X^{-1}{)}_{i}^{\nu }$ . In matrix notation, we write $(T{)}_{ij}^{ab}=(YX^{-1}{)}_{ij}^{ab}=t_{ij}^{ab}$ . Using this identity with eq 11, we have

10 $$\left(\begin{array}{ll} f & \mathrm{U̅}\\ U & K^{>,2p1h}+C^{>,2p1h} \end{array}\right)\left(\begin{array}{l} l\\ T \end{array}\right)=\left(\begin{array}{l} l\\ T \end{array}\right)XEX^{-1}$$

These simultaneous equations give the extended Fock operator and the self-energy Riccati equation:

11a FX=XE

11b $$U+\left(K^{>,2p1h}+C^{>,2p1h}\right)T-TF=0$$

where **F** = **f** + **U̅T** is the extended Fock operator. In explicit matrix notation, the extended Fock operator is written as

12 $$F_{ij}=\epsilon_{i}\delta_{ij}+\frac{1}{2}\underset{kab}{\sum }\left\langle ik\left|\right|ab\right\rangle t_{jk}^{ab}$$

The extended Fock operator is exactly of the form of the upper left block in IP-EOM-CCD theory: *F_ij_* = −⟨Φ*_j_* |*H̅_N_* |Φ*_i_* ⟩, where ${\mathrm{H̅}}_{N}=e^{-T_{2}}He^{T_{2}}-E_{0}^{\mathrm{CC}}$ is the normal-ordered CCD similarity transformed Hamiltonian.^15,16,29^ Equation 12 is also found by neglecting the *T*_1_ amplitudes appearing in refs 30–33 . Using the coupling and interaction matrix elements defined above in eqs 5 and 7, the self-energy Riccati equation is explicitly written as

13 $$\begin{array}{l} \left\langle ab\left|\right|ij\right\rangle +\frac{1}{2}\underset{kl}{\sum }(t_{kl}^{ab}{)}_{\mathrm{MP}2}\left\langle ij\left|\right|kl\right\rangle -\underset{kc}{\sum }\left\langle kb\left|\right|ic\right\rangle (t_{kj}^{ac}{)}_{\mathrm{MP}2}\\ +\underset{kc}{\sum }\left\langle ka\left|\right|ic\right\rangle (t_{kj}^{bc}{)}_{\mathrm{MP}2}+\Delta_{ij}^{ab}t_{ij}^{ab}+\frac{1}{2}\underset{cd}{\sum }\left\langle ab\left|\right|cd\right\rangle t_{ij}^{cd}\\ -\underset{kc}{\sum }\left\langle kb\left|\right|jc\right\rangle t_{ik}^{ac}+\underset{kc}{\sum }\left\langle ka\left|\right|jc\right\rangle t_{ik}^{bc}\\ -\frac{1}{2}\underset{klcd}{\sum }t_{ik}^{cd}\left\langle kl\left|\right|cd\right\rangle t_{jl}^{ab}=0 \end{array}$$

The self-energy Riccati equation as defined in eq 14 represents a subset of the full CCD equations:^34^

14 $$\begin{array}{l} \left\langle ab\left|\right|ij\right\rangle +\Delta_{ij}^{ab}t_{ij}^{ab}+\frac{1}{2}\underset{cd}{\sum }\left\langle ab\left|\right|cd\right\rangle t_{ij}^{cd}+\frac{1}{2}\underset{kl}{\sum }\left\langle ij\left|\right|kl\right\rangle t_{kl}^{ab}\\ -\underset{kc}{\sum }\left(\left\langle kb\left|\right|jc\right\rangle t_{ik}^{ac}-\left\langle ka\left|\right|jc\right\rangle t_{ik}^{bc}+\left\langle ak\left|\right|ci\right\rangle t_{jk}^{bc}-\left\langle bk\left|\right|ci\right\rangle t_{jk}^{ac}\right)\\ \underset{klcd}{\sum }\left\langle kl\left|\right|cd\right\rangle (\frac{1}{4}t_{kl}^{ab}t_{ij}^{cd}-\frac{1}{2}\left(t_{ik}^{ab}t_{jl}^{cd}+t_{ik}^{cd}t_{jl}^{ab}+t_{ij}^{ac}t_{kl}^{bd}+t_{ij}^{bd}t_{kl}^{ac}\right)+\left(t_{ik}^{ac}t_{jl}^{bd}+t_{ik}^{bd}t_{jl}^{ac}\right))=0 \end{array}$$

As can be seen by analysis of eq 17, the self-energy CCD amplitude equations given in eq 16 are missing several additional terms. These missing contributions include the particle–particle ladder terms, the full antisymmetrized ring diagrams as well as the six additional quadratic terms. Another difference is that only the MP2 doubles amplitudes appear in the hole–hole ladder and ring terms of the self-energy amplitude equations. These effectively represent a first iteration of the CCD equations using the MP2 amplitudes as an initial guess.

Our approach shares several similarities to the rCCD amplitude equations, first derived in ref 35. However, the rCCD amplitude equations are given by^6,35–38^

15 $$\left\langle ab\left|\right|ij\right\rangle +\Delta_{ij}^{ab}t_{ij}^{ab}-\underset{kc}{\sum }\left\langle kb\left|\right|jc\right\rangle t_{ik}^{ac}-\underset{kc}{\sum }\left\langle ka\left|\right|ic\right\rangle t_{jk}^{bc}+\underset{klcd}{\sum }t_{ik}^{ac}\left\langle kl\left|\right|cd\right\rangle t_{jl}^{bd}=0$$

and originate from the RPA eigenvalue problem. The RPA approximation only contains ring diagram contributions to the CCD equations that are not antisymmetrized with respect to exchange of the external indices.

It should be noted that the MP2 self-energy approximation corresponds to taking the interaction matrices to be $K_{iab,jcd}^{>,\mathrm{MP}2}=\left(\epsilon_{a}+\epsilon_{b}-\epsilon_{i}\right)\left(\delta_{ac}\delta_{bd}-\delta_{ad}\delta_{bc}\right)\delta_{ij}$ . This yields the effective doubles amplitude equations for the particle-hole-time decoupled MP2 self-energy:

16 $$\left\langle ab\left|\right|ij\right\rangle +\Delta_{ij}^{ab}t_{ij}^{ab}-\frac{1}{2}\underset{klcd}{\sum }t_{ik}^{cd}\left\langle kl\left|\right|cd\right\rangle t_{jl}^{ab}=0$$

We now demonstrate how to obtain the full CCD equations from the electronic self-energy. To do so we must transform the coupling and interaction matrices to become self-consistently dependent on the corresponding amplitude solutions. For the coupling matrices, eq 5, this simply corresponds to replacing all MP2 amplitudes with the exact doubles amplitudes to be determined: $(t_{ij}^{ab}{)}_{\mathrm{MP}2}\to t_{ij}^{ab}$ . This results in the “self-consistent” coupling matrix elements:

17 $$U_{abj,p}^{\mathrm{sc}}=\left\langle ab\left|\right|pj\right\rangle +\frac{1}{2}\underset{kl}{\sum }t_{kl}^{ab}\left\langle pj\left|\right|kl\right\rangle -\underset{kc}{\sum }\left\langle kb\left|\right|pc\right\rangle t_{kj}^{ac}+\underset{kc}{\sum }\left\langle ka\left|\right|pc\right\rangle t_{kj}^{bc}$$

These coupling matrix elements can be viewed in terms of a self-consistent Green’s function theory whereby the solution is updated iteratively. For the interaction matrices, the following self-consistent updates are necessary:

18a $$\epsilon_{i}\delta_{ij}\to \epsilon_{i}\delta_{ij}+\frac{1}{2}\underset{kcd}{\sum }\left\langle ik\left|\right|cd\right\rangle t_{jk}^{cd}$$

18b $$\epsilon_{a}\delta_{ab}\to \epsilon_{a}\delta_{ab}-\frac{1}{2}\underset{ijc}{\sum }\left\langle ij\left|\right|bc\right\rangle t_{ij}^{ca}$$

18c $$\left\langle ab\left|\right|cd\right\rangle \to \left\langle ab\left|\right|cd\right\rangle +\frac{1}{2}\underset{kl}{\sum }\left\langle kl\left|\right|cd\right\rangle t_{kl}^{ab}$$

18d $$\left\langle ja\left|\right|bi\right\rangle \to \left\langle ja\left|\right|bi\right\rangle +\underset{kc}{\sum }\left\langle jk\left|\right|bc\right\rangle t_{ik}^{ac}$$

Including these additional terms in the interaction matrices, $\left(K_{iab,jcd}^{>,2p1h}+C_{iab,jcd}^{>,2p1h}\right)$ , (see eq 7) that enter the self-energy Riccati (eq 14) gives rise to the six additional quadratic terms required to generate the full CCD equations given in eq 17. The additional terms, generated by the replacements given in eqs 18, can be viewed as self-consistent fourth-order electronic self-energy diagrams that contain contributions in the 2p1h/2h1p excitation space (see Figure 2 and) that are present in the ADC(4) construction.^17^ However, in the perturbative ADC(4) construction their contribution is restricted to contain MP2 doubles amplitudes only and necessarily requires the expansion of the Dyson supermatrix to include 3p2h/3h2p intermediate state configurations.^17,20^ The self-energy Riccati equation, eq 14, resulting from the replacements made in eqs 20 and eqs 18 to the coupling and interaction matrices gives the full CCD amplitude equations. These results demonstrate that the full CCD equations can be viewed as an infinite partial summation through fourth-order of perturbation theory in the electronic self-energy when restricted to 2p1h/2h1p excitation character of the Intermediate State Configurations. The Feynman-Goldstone diagrams for the particle-hole-time decoupled self-energy that generate the full CCD amplitude equations are given in Appendix A. To the best of our knowledge, this is the first time that the *complete* CCD amplitude equations have been derived within the Green’s function formalism.

**Figure 2:**
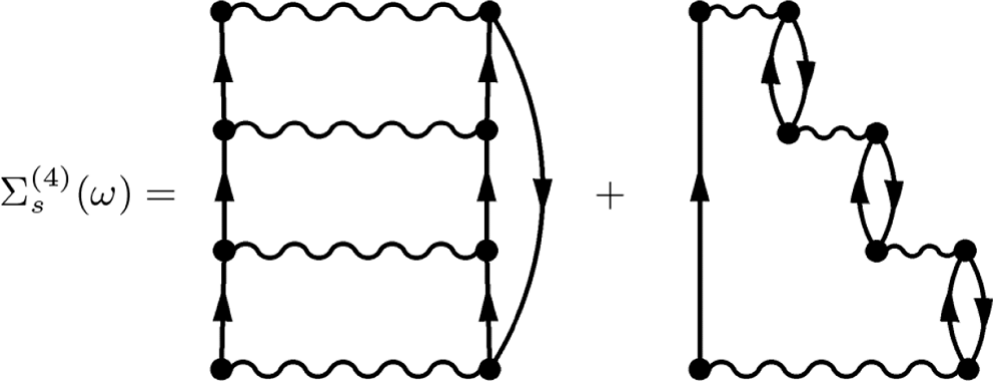
Fourth-order one-particle irreducible skeleton electronic self-energy diagrams contained in the full CCD equations when their contribution is included in the set of 2p1h/2h1p interaction matrices.

It is well established that the singles amplitudes, $\left\{t_{i}^{a}\right\}$ , can be formally eliminated by the use of Brueckner orbitals whereby the spin–orbitals are determined simultaneously with the optimization of the cluster amplitudes.^29,39–42^ This orbital rotation determined by the singles amplitude equations is termed Brueckner coupled-cluster theory (BCC). Therefore, upon rotation to the Brueckner orbitals the derivation of the doubles amplitude equations presented above is unchanged. Additionally, the formalism presented in this work can also be extended to derive the higher body BCCDT amplitude equations by retention and identification of the correct time-ordered self-energy diagrams contained in the higher-order ADC­(*n*) approximations.

The ground state correlation energy is found from the relation

19 $$\begin{array}{ll} E_{0}^{N} & =\frac{1}{2}trXEX^{-1}+\frac{1}{2}tr\left[h\right]\\ & =E_{0}^{HF}+\frac{1}{2}tr\left[\mathrm{U̅}T\right]\\ & =E_{0}^{HF}+\frac{1}{4}\underset{ijab}{\sum }\left\langle ij\left|\right|ab\right\rangle t_{ij}^{ab} \end{array}$$

where we have used the identity: $XEX^{-1}=F$ from eq 15 to go from the first line to second line of eq 25. This expression is exactly equivalent to that obtained from CCD theory. eq 19 demonstrates that we can obtain correlation energies from Green’s function theory that can be cast exactly in terms of CCD theory.

The doubles amplitude equations generated by the particle-hole-time decoupled self-energy for the virtual block can be found in Appendix B and are identical through particle-hole symmetry.

The relationship to the IP-EOM-CCD eigenvalue problem can be seen by coupling the self-consistent backward-time particle-hole-time decoupled self-energy (see eq 10 and Appendix B) to the occupied-occupied block of the self-energy while maintaining the particle-hole separation. This coupling is necessary to account for the negation of the full time-orderings of the coupling matrices of the forward-time particle-hole-time decoupled self-energy (see eq 9) in order to obtain accurate approximations for the removal excitation energies. Inclusion of these terms results in equations that are analogous to those of IP-EOM-CCD theory.^31–33,43–45^ As particle-hole separation is maintained, the resulting self-energy approximation is still of the non-Dyson form. Therefore, the presence of the lambda de-excitation amplitudes, {λ_μ_} required to construct the left eigenstate of *H̅*, that appear in refs 15 and 16 in the diagrammatic coupled-cluster self-energy do not appear in this work. As the non-Dyson approximation is kept, the connection can be explicitly made to IP/EA-EOM-CCD theory, where the particle-hole sectors are decoupled. The coupling of backward-time components to the particle-hole-time decoupled self-energy of the occupied-occupied block gives the additional particle-hole decoupled self-energy contribution

20 $${\mathrm{\Sigma ̅}}_{ij}^{B}\left(\omega \right)=\underset{\begin{array}{l} k>la\\ m>nb \end{array}}{\sum }V_{i,kla}^{\mathrm{sc}}\times {\left(\left(\omega -i\eta \right)l-\left({\mathrm{K̅}}^{<,2h1p}+{\mathrm{C̅}}^{<,2h1p}\right)\right)}_{kla,mnb}^{-1}{\mathrm{V̅}}_{mnb,j}^{†}$$

where we define the transformed coupling elements from the self-consistent coupling matrices given in Appendix B and reproduced here for clarity:

21a $$V_{i,kla}^{\mathrm{sc}}=\left\langle ia\left|\right|kl\right\rangle +\underset{cm}{\sum }\left\langle im\left|\right|kc\right\rangle t_{ml}^{ca}-\underset{cm}{\sum }\left\langle im\left|\right|lc\right\rangle t_{mk}^{ca}+\frac{1}{2}\underset{cd}{\sum }\left\langle ia\left|\right|cd\right\rangle t_{kl}^{cd}$$

with

21b $${\mathrm{V̅}}_{mnb,j}^{†}=\left\langle mn\left|\right|jb\right\rangle$$

These coupling matrix elements are exactly equal to the blocks of the CCD similarity transformed Hamiltonian: $V_{i,kla}=\left\langle \Phi_{kl}^{a}\left|{\mathrm{H̅}}_{N}\right|\Phi_{i}\right\rangle$ and ${\mathrm{V̅}}_{mnb,j}^{†}=\left\langle \Phi_{j}\left|{\mathrm{H̅}}_{N}\right|\Phi_{mn}^{b}\right\rangle$ .^10,34^

The self-consistent interaction matrices are exactly those appearing in Appendix B and are written compactly as^15,16^

22 $$\begin{array}{ll} \left({\mathrm{K̅}}_{ija,klb}^{<,2h1p}+{\mathrm{C̅}}_{ija,klb}^{<,2h1p}\right) & =F_{ik}\delta_{ab}\delta_{jl}+F_{jl}\delta_{ab}\delta_{ik}-F_{ba}\delta_{ik}\delta_{jl}\\ & -F_{il}\delta_{ab}\delta_{jk}-F_{jk}\delta_{ab}\delta_{il}+F_{ba}\delta_{il}\delta_{jk}\\ & +\chi_{ib,al}\delta_{jk}+\chi_{jb,ak}\delta_{il}-\chi_{ij,kl}\delta_{ab}\\ & -\chi_{ib,ak}\delta_{jl}-\chi_{jb,al}\delta_{ik} \end{array}$$

We maintain particle-hole separability such that ${\mathrm{\Sigma ̅}}_{ia}={\mathrm{\Sigma ̅}}_{ai}=0$ . The interaction matrices are almost identical to the matrix elements of the CCD similarity transformed Hamiltonian in the basis of 2h1p determinants: $\left({\mathrm{K̅}}_{ija,klb}^{<,2h1p}+{\mathrm{C̅}}_{ija,klb}^{<,2h1p}\right)\approx -\left\langle \Phi_{kl}^{b}\left|{\mathrm{H̅}}_{N}\right|\Phi_{ij}^{a}\right\rangle$ . However, the explicit three-body interaction, $\chi_{ijb,kal}$ , that arises in IP-EOM-CCD theory is not present in eq 29.^15,16,31–33,46^ The result of this coupling is the effective particle-hole decoupled quasiparticle Hamiltonian

23 $${Ĥ}_{ij}^{\mathrm{IP‐EOM‐CCD}}\left(\omega \right)=F_{ij}+{\mathrm{\Sigma ̅}}_{ij}^{B}\left(\omega \right)$$

from which we have the upfolded supermatrix representation:

24 $${\mathrm{D̅}}^{\mathrm{IP‐EOM‐CCD}}=\left(\begin{array}{ll} f+\mathrm{U̅}T & V^{\mathrm{sc}}\\ {\mathrm{V̅}}^{†} & {\mathrm{K̅}}^{<,2h1p}+{\mathrm{C̅}}^{<,2h1p} \end{array}\right)$$

Explicitly, we can write this supermatrix in the suggestive form

25 $${\mathrm{D̅}}^{\mathrm{IP‐EOM‐CCD}}=\left(\begin{array}{ll} -\left\langle \Phi_{j}\left|{\mathrm{H̅}}_{N}\right|\Phi_{i}\right\rangle & \left\langle \Phi_{kl}^{a}\left|{\mathrm{H̅}}_{N}\right|\Phi_{i}\right\rangle \\ \left\langle \Phi_{j}\left|{\mathrm{H̅}}_{N}\right|\Phi_{mn}^{b}\right\rangle & -\left\langle \Phi_{mn}^{b}\left|{\mathrm{H̅}}_{N}\right|\Phi_{kl}^{a}\right\rangle \end{array}\right)$$

remembering that the interaction matrices (bottom right block of the supermatrix) do not include the three-body CC interaction. Supermatrices of the form of eqs 31/32 have structures that are analogous to the IP-EOM-CCD supermatrix as demonstrated in refs 15 and 16. The eigenvalues of **D̅**^IP‑EOM‑CCD^ provide access to approximate electron removal energies. However, the residues of the single-particle Green’s function are not obtained within this approximation as the particle-hole sectors remain decoupled. The relationship to EA-EOM-CCD theory can be derived in an entirely analogous manner by coupling the forward time self-energy contributions to the virtual states (see eq 10) while maintaining particle-hole separability.

## 4 Application to a Model System: Exact Solution of the Hubbard Dimer

We apply the formalism presented in this work to the exactly solvable Hubbard dimer at half filling. The Hubbard model is parametrized by the ratio $\frac{U}{t}$ , which provides a measure of the “correlation strength”. The parameters *U* and *t* represent the on-site repulsion and the coupling strength between nearest neighbor sites, respectively.^47^ The CCD amplitude equations are exact for the Hubbard dimer and their quadratic Riccati equation yields two solutions:

26 $$(t_{ii}^{aa}{)}_{\pm }=\frac{4t\pm c}{U}$$

where $c=\sqrt{U^{2}+16t^{2}}$ . The CCD amplitude is the negative solution, $(t_{ii}^{aa}{)}_{-}$ , which gives the exact ground state correlation energy: $E_{c}=\frac{U}{2}(t_{ii}^{aa}{)}_{-}=2t-\frac{c}{2}$ .

In ref 48, we demonstrated that the second-order MP2 self-energy is exact for the Hubbard dimer. From the MP2 self-energy, the corresponding Dyson supermatrix for this system splits into the following eigenvalue problems for the occupied and virtual blocks respectively

27a $$\left(\begin{array}{ll} \epsilon_{i} & \frac{U}{2}\\ \frac{U}{2} & 2\epsilon_{a}-\epsilon_{i} \end{array}\right)\left(\begin{array}{l} X\\ Y \end{array}\right)=\left(\begin{array}{l} X\\ Y \end{array}\right)\varepsilon^{h}$$

27b $$\left(\begin{array}{ll} \epsilon_{a} & \frac{U}{2}\\ \frac{U}{2} & 2\epsilon_{i}-\epsilon_{a} \end{array}\right)\left(\begin{array}{l} \mathrm{X̃}\\ Ỹ \end{array}\right)=\left(\begin{array}{l} \mathrm{X̃}\\ Ỹ \end{array}\right)\varepsilon^{p}$$

where $\epsilon_{i/a}=\frac{U}{2}\mp t$ are the HF reference energies of the occupied/virtual states, respectively.

Multiplying eq 34 by *X* ^–1^, we obtain the extended Fock operator and self-energy Riccati equation as

28a $$\epsilon_{i}+\frac{U}{2}t_{ii}^{aa}=\varepsilon^{h}$$

28b $$\frac{U}{2}+2\left(\epsilon_{a}-\epsilon_{i}\right)t_{ii}^{aa}-\frac{U}{2}(t_{ii}^{aa}{)}^{2}=0$$

where we have used the identity: $t_{ii}^{aa}=Y\cdot X^{-1}$ . The self-energy Riccati equation, eq 36, is the exact CCD amplitude equation:

29 $$\frac{U}{2}(t_{ii}^{aa}{)}^{2}-4t\left(t_{ii}^{aa}\right)-\frac{U}{2}=0$$

and yields two solutions: $(t_{ii}^{aa}{)}_{\pm }=\frac{1}{U}\left(4t\pm c\right)$ . The corresponding hole eigenvalues and supereigenvectors of the quasiparticle and satellite solutions are found by substitution of the CCD amplitude solutions, $(t_{ii}^{aa}{)}_{\pm }$ , into eq 36 to give

30a $$\varepsilon_{\mathrm{QP}}^{h}=\epsilon_{i}+\frac{U}{2}(t_{ii}^{aa}{)}_{-};,v_{\mathrm{QP}}^{h}=\left(\begin{array}{l} 1\\ (t_{ii}^{aa}{)}_{-} \end{array}\right)$$

30b $$\varepsilon_{\mathrm{sat}}^{h}=\epsilon_{i}+\frac{U}{2}(t_{ii}^{aa}{)}_{+};,v_{\mathrm{sat}}^{h}=\left(\begin{array}{l} 1\\ (t_{ii}^{aa}{)}_{+} \end{array}\right)$$

The CCD amplitude is the lower component of the $v_{\mathrm{QP}}^{h}$ vector of the hole quasiparticle solution: $(t_{ii}^{aa}{)}_{-}=\frac{4t-c}{U}$ . From eq 4, the inverse of the norm of the vector is exactly the hole quasiparticle renormalization factor: $Z_{h}^{\mathrm{QP}}={\left(v_{\mathrm{QP}}^{h†}v_{\mathrm{QP}}^{h}\right)}^{-1}={\left(1+(t_{ii}^{aa}{)}_{-}^{2}\right)}^{-1}=\frac{1}{2}+\frac{2t}{c}$ . The hole satellite solution contains a lower component of the eigenvector, $v_{\mathrm{sat}}^{h}$ , that is exactly the positive CCD amplitude solution: $(t_{ii}^{aa}{)}_{+}=\frac{1}{U}\left(4t+c\right)$ , with the satellite renormalization factor as $Z_{h}^{\mathrm{sat}}={\left(1+(t_{ii}^{aa}{)}_{+}^{2}\right)}^{-1}=\frac{1}{2}-\frac{2t}{c}$ . As expected, the renormalization factor for the quasiparticle is larger than that of the satellite, $Z_{h}^{\mathrm{QP}}>Z_{h}^{\mathrm{sat}}$ . Using eq 25 for the ground state correlation energy gives the exact ground state correlation energy of the Hubbard dimer.^49–52^

For the particle solutions, the amplitude equations are directly connected to the hole solutions via particle-hole symmetry (see Appendix B). The corresponding particle eigenvalues and supereigenvectors of the quasiparticle and satellite solutions are given by

31a $$\varepsilon_{\mathrm{QP}}^{p}=\epsilon_{a}-\frac{U}{2}(t_{ii}^{aa}{)}_{-};,v_{\mathrm{QP}}^{p}=\left(\begin{array}{l} 1\\ -(t_{ii}^{aa}{)}_{-}^{*} \end{array}\right)$$

31b $$\varepsilon_{\mathrm{sat}}^{p}=\epsilon_{a}-\frac{U}{2}(t_{ii}^{aa}{)}_{+};,v_{\mathrm{sat}}^{p}=\left(\begin{array}{l} 1\\ -(t_{ii}^{aa}{)}_{+}^{*} \end{array}\right)$$

where we have used the relation from Appendix B: ${\mathrm{t̃}}_{ii}^{aa}=Ỹ\cdot {\mathrm{X̃}}^{-1}=-(t_{ii}^{aa}{)}^{*}$ . The quasiparticle renormalization factor is again $Z_{p}^{\mathrm{QP}}={\left(1+(t_{ii}^{aa}{)}_{-}^{2}\right)}^{-1}$ . The satellite particle solution gives the lower component of the $v_{\mathrm{sat}}^{p}$ vector as the negative of the positive CCD solution: $-(t_{ij}^{ab}{)}_{+}$ , with the renormalization factor given by $Z_{p}^{\mathrm{sat}}={\left(1+(t_{ii}^{aa}{)}_{+}^{2}\right)}^{-1}$ . Again, the renormalization factor of the quasiparticle is larger than that of the satellite, $Z_{p}^{\mathrm{QP}}>Z_{p}^{\mathrm{sat}}$ . Using these expressions is it simple to verify that $Z_{h/p}^{\mathrm{QP}}+Z_{h/p}^{\mathrm{sat}}=1$ , as required by the sum rule.

Substituting the expression for the CCD amplitudes (eq 33) obtained from the self-energy Riccati equation (eq 37) into eqs 30 and 31, we see that the particle and hole quasiparticle/satellite energies and renormalization factors are exact for the Hubbard dimer.^48,53–55^ The satellite and quasiparticle energies are exactly of the form of the extended Fock operators defined in eq 15 and B3. These results concretely demonstrate that different CCD amplitude solutions can correspond to satellite excited states resulting from particle removal and addition processes. From our formulation, we can explicitly write the exact single-particle Greens function in terms of the CCD amplitudes:

32a $$G_{ii}\left(\omega \right)={\left(1+(t_{ii}^{aa}{)}_{-}^{2}\right)}^{-1}\frac{1}{\omega -(\epsilon_{i}+\frac{U}{2}\left(t_{ii}^{aa}{)}_{-}\right)-i\eta }+{\left(1+(t_{ii}^{aa}{)}_{+}^{2}\right)}^{-1}\frac{1}{\omega -(\epsilon_{i}+\frac{U}{2}\left(t_{ii}^{aa}{)}_{+}\right)+i\eta }$$

32b $$G_{aa}\left(\omega \right)={\left(1+(t_{ii}^{aa}{)}_{-}^{2}\right)}^{-1}\frac{1}{\omega -(\epsilon_{a}-\frac{U}{2}\left(t_{ii}^{aa}{)}_{-}\right)+i\eta }+{\left(1+(t_{ii}^{aa}{)}_{+}^{2}\right)}^{-1}\frac{1}{\omega -(\epsilon_{a}-\frac{U}{2}\left(t_{ii}^{aa}{)}_{+}\right)-i\eta }$$

In Figures 3 and 4, we plot the poles and weights of the exact Green’s function given in eqs 32 against the HF and *GW* approximations obtained from ref 48. In Figure 3, we see that the quasiparticle *GW* solutions are close to the exact quasiparticle solutions obtained within our formalism for values of $\frac{U}{t}$ up to 3. Beyond this point, the quasiparticle *GW* solutions begin to diverge away from the exact results as the essential correlated physics of electron localization is not recovered. In Figure 3, we also see that the *GW* satellite solutions are completely qualitatively wrong. This is a result of the fact that the *GW* approximation cannot handle the increasing multireference character of the underlying ground and excited state wave functions as the correlation strength $\frac{U}{t}$ increases.^48,56^

**Figure 3:**
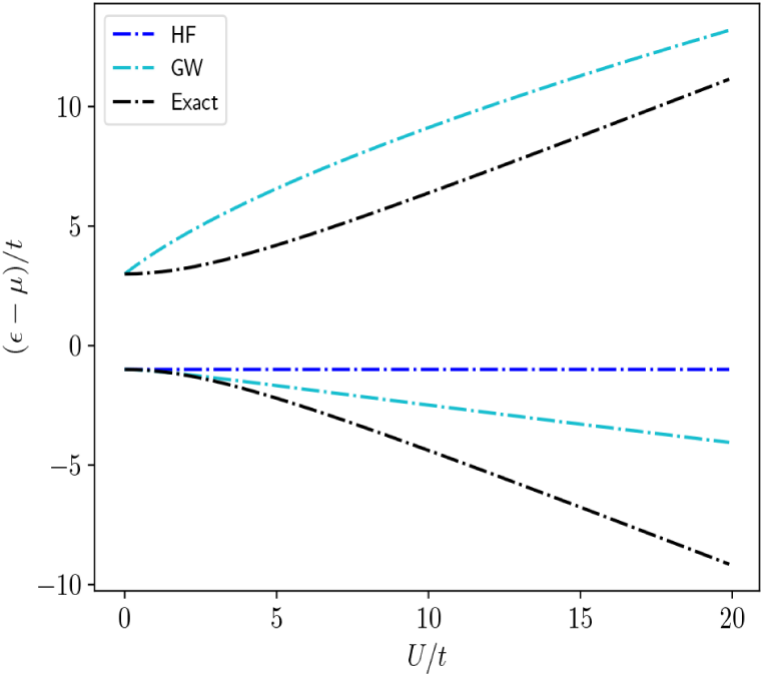
Hole quasiparticle and satellite energies of the Hubbard dimer as a function of $\frac{U}{t}$ relative to the chemical potential, $\mu =\frac{U}{2}$ . The particle states are related to the hole states by particle-hole symmetry.

**Figure 4:**
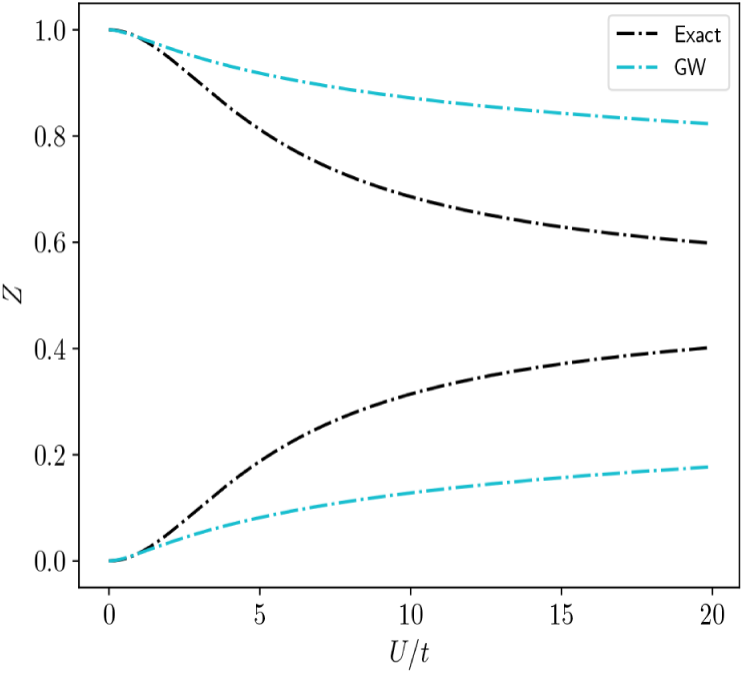
$Z_{p/h}^{\mathrm{QP}/\mathrm{sat}}$ renormalization factors for the Hubbard dimer as a function of $\frac{U}{t}$ .

In Figure 4, we plot the quasiparticle and satellite renormalization factors as a function of $\frac{U}{t}$ . We find that the *GW* solutions diverge quickly from the exact results even in the weakly correlated regime as a result of the strong multireference character of the ground and excited state wave functions.^48^ In Figure 5, we show the exact and *GW* spectral functions, evaluated at $\frac{U}{t}=7$ , obtained from the trace of the imaginary part of the corresponding Green’s function. From analysis of the spectral function, we clearly see the qualitatively incorrect satellite peaks and strengths obtained from the *GW* approximation relative to the exact solution obtained from eqs 32. Additionally, we see that the *GW* quasiparticle peaks are in qualitatively better agreement with the exact solution, but thattheir relative peak strength is larger as a result of the underestimation of the satellite weights.

**Figure 5:**
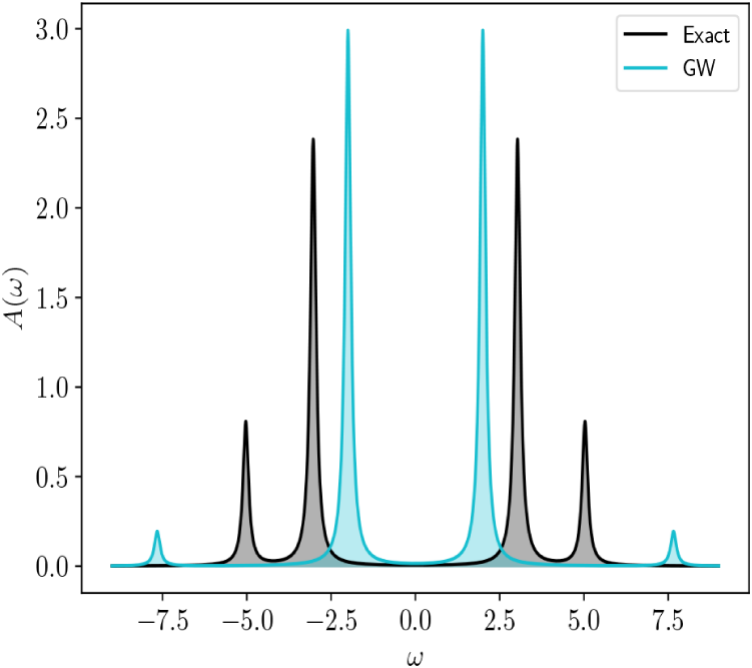
Exact and *GW* spectral functions obtained for the Hubbard dimer at $\frac{U}{t}=7$ . The exact spectral function is obtained from eqs 32 as A(ω)=1π∑p|Im⁡Gpp(ω)| .

## 5 Conclusions and Outlook

In summary, we have firmly demonstrated the connection between the electronic self-energy and coupled-cluster doubles theory. To do so, we decouple the particle-hole as well as forward- and backward-time sectors of the electronic self-energy. Our formal insights have demonstrated that CCD theory represents a self-consistent, infinite-order summation of fourth-order electronic self-energy diagrams restricted to the space of 2p1h/2h1p excitations. The relationship to the IP/EA-EOM-CCD eigenvalue problem can be revealed be coupling reverse-time self-energy contributions to either the occupied or virtual sectors while maintaining particle-hole separability. Finally, using the formalism developed in this paper, we reconstruct the exact Green’s function of the Hubbard dimer in terms of the CCD amplitudes. We hope that our findings will help stimulate renewed work toward combining Green’s function and coupled-cluster theories for ground and excited state many-body correlation. Further directions of this work would be to develop a Hermitian ADC(3) self-energy approximation that contains the exact CCD amplitudes obtained from a ground state coupled-cluster calculation. This would allow for the use of ground state coupled-cluster amplitudes, that contain infinite-order partial summations of the forward-time self-energy diagrams derived in this work, within the Hermitian structure of the conventional Green’s function formalism to obtain charged excitation spectra. In principle, the relationships identified here can also be used to generate novel CCD approximations from the *GW* approximation. The extension of the analysis presented here to the derivation of the coupled doubles and triples amplitude equations is reserved for future work.

## Figures and Tables

**Figure 6:**
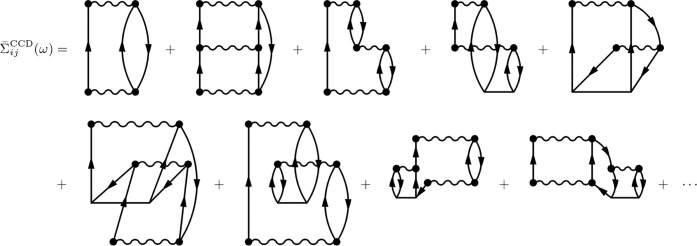
2p1h/2h1p excitation-restricted Feynman-Goldstone one-particle irreducible electronic particle-hole-time decoupled self-energy diagrams that generate the CCD amplitude equations when summed to infinite order by the Dyson supermatrix. The final four diagrams depicted are fourth-order self-energy contributions. The solid line represents the CCD amplitude, $t_{ij}^{ab}$ .

## Particle-hole-time decoupled electronic self-energy diagrams contained in CCD theory

The set of one-particle irreducible particle-hole-time decoupled self-energy diagrams that generate the exact CCD amplitude equations are shown in Figure 6. In Figure 6, we introduce the self-consistent notation, whereby the interaction and coupling matrices are now dependent on the solution of the CCD amplitudes. This amounts to replacing the MP2 amplitudes that are be obtained from the perturbative electronic self-energy diagrams by the full CCD amplitudes: $(t_{ij}^{ab}{)}_{\mathrm{MP}2}\to t_{ij}^{ab}$ .

Downfolding eq 10, using the modified self-consistent coupling and interaction matrices that depend on the exact CCD amplitudes (eqs 17 and 18) leads to the expression

A1
$${\mathrm{\Sigma ̅}}_{ij}^{\mathrm{CCD}}\left(\omega \right)=\frac{l}{4}\underset{\begin{array}{l} kab\\ lcd \end{array}}{\sum }{\mathrm{U̅}}_{i,abk}{\left(\left(\omega +i\eta \right)l-\left({\mathrm{K̅}}^{>,2p1h}+{\mathrm{C̅}}^{>,2p1h}\right)\right)}_{kab,lcd}^{-1}U_{cdl,j}^{\mathrm{sc}}$$

where the interaction matrix elements, $\left({\mathrm{K̅}}_{kab,jcd}^{>,2p1h}+{\mathrm{C̅}}_{kab,jcd}^{>,2p1h}\right)$ , are obtained by using eqs 18 to update the interaction matrices defined in eq 7. The self-consistent coupling matrix elements, $U_{cdl,j}^{\mathrm{sc}}$ , are defined in eq 17. The infinite series of self-energy diagrams contained in eq A1 is presented in Figure 6.

## Doubles amplitudes from the particle-hole-time decoupled self-energy of the virtual states

For the virtual block of the particle-hole-time decoupled self-energy, we have the following supermatrix eigenvalue problem

$$\left(\begin{array}{ll} f & \mathrm{V̅}\\ V^{†} & K^{<,2h1p}+C^{<,2h1p} \end{array}\right)\left(\begin{array}{l} \mathrm{X̃}\\ Ỹ \end{array}\right)=\left(\begin{array}{l} \mathrm{X̃}\\ Ỹ \end{array}\right)E\sim$$
B1

Defining the effective doubles amplitudes as $\mathrm{T̃}=Ỹ{\mathrm{X̃}}^{-1}$ we have the extended Fock operator and self-energy Riccati equation

B2a
FX̃=X̃Ẽ

B2b
$$V^{†}+\left(K^{<,2h1p}+C^{<,2h1p}\right)\mathrm{T̃}-\mathrm{T̃}F=0$$

B3
$$\begin{array}{ll} F_{ab} & =\epsilon_{a}\delta_{ab}+\frac{1}{2}\underset{klc}{\sum }\left\langle ac\left|\right|kl\right\rangle {\mathrm{t̃}}_{kl}^{bc}\\ & =\epsilon_{a}\delta_{ab}-\frac{1}{2}\underset{klc}{\sum }\left\langle kl\left|\right|bc\right\rangle t_{kl}^{ac} \end{array}$$

B4
$$\begin{array}{l} \left\langle ij\left|\right|ab\right\rangle +\frac{1}{2}\underset{cd}{\sum }\left\langle ab\left|\right|cd\right\rangle (t_{ij}^{cd}{)}_{\mathrm{MP}2}+\underset{kc}{\sum }\left\langle ka\left|\right|jc\right\rangle (t_{ik}^{bc}{)}_{\mathrm{MP}2}\\ -\underset{kc}{\sum }\left\langle ak\left|\right|ci\right\rangle (t_{jk}^{bc}{)}_{\mathrm{MP}2}-\Delta_{ij}^{ab}{\mathrm{t̃}}_{ij}^{ab}-\frac{1}{2}\underset{kl}{\sum }\left\langle ij\left|\right|kl\right\rangle {\mathrm{t̃}}_{kl}^{ab}\\ +\underset{kc}{\sum }\left\langle jc\left|\right|kb\right\rangle {\mathrm{t̃}}_{ik}^{ac}-\underset{kc}{\sum }\left\langle ic\left|\right|kb\right\rangle {\mathrm{t̃}}_{jk}^{ac}\\ -\frac{1}{2}\underset{klcd}{\sum }{\mathrm{t̃}}_{kl}^{ac}\left\langle cd\left|\right|kl\right\rangle {\mathrm{t̃}}_{ij}^{bd}=0 \end{array}$$

B5
$$\begin{array}{l} \left\langle ab\left|\right|ij\right\rangle +\frac{1}{2}\underset{cd}{\sum }\left\langle ab\left|\right|cd\right\rangle (t_{ij}^{cd}{)}_{\mathrm{MP}2}+\underset{kc}{\sum }\left\langle ka\left|\right|jc\right\rangle (t_{ik}^{bc}{)}_{\mathrm{MP}2}\\ -\underset{kc}{\sum }\left\langle ak\left|\right|ci\right\rangle (t_{jk}^{bc}{)}_{\mathrm{MP}2}+\Delta_{ij}^{ab}t_{ij}^{ab}+\frac{1}{2}\underset{kl}{\sum }\left\langle ij\left|\right|kl\right\rangle t_{kl}^{ab}\\ -\underset{kc}{\sum }\left\langle kb\left|\right|jc\right\rangle t_{ik}^{ac}+\underset{kc}{\sum }\left\langle kb\left|\right|ic\right\rangle t_{jk}^{ac}\\ -\frac{1}{2}\underset{klcd}{\sum }t_{kl}^{ac}\left\langle kl\left|\right|cd\right\rangle t_{ij}^{bd}=0 \end{array}$$

where **F** = **f** + **V̅T̃**. We immediately apply the constraint of particle-hole symmetry: ${\mathrm{t̃}}_{ij}^{ab}=-(t_{ij}^{ab}{)}^{*}$ . The corresponding extended Fock operator in the virtual space is then given bywhere we have used the constraint of hermiticity: $F_{ab}=F_{ba}^{*}$ . This expression is exactly that obtained from CCD theory for the extended Fock operator.^15,16^ The amplitude equations becomewhich can be re-written by imposing the particle-hole symmetry constraint as To generate the full CCD equations from the virtual block, we must self-consistently update the coupling and interaction matrices as follows. First, we modify the coupling matrices to be self-consistently determined from the doubles amplitude solutions:

B6
$$V_{p,kla}^{\mathrm{sc}}=\left\langle pa\left|\right|kl\right\rangle +\underset{cm}{\sum }\left\langle pm\left|\right|kc\right\rangle t_{ml}^{ca}-\underset{cm}{\sum }\left\langle pm\left|\right|lc\right\rangle t_{mk}^{ca}+\frac{1}{2}\underset{cd}{\sum }\left\langle pa\left|\right|cd\right\rangle t_{kl}^{cd}$$

This is analogous to the procedure carried out for the occupied block. Then we update the interaction matrices between the 2h1p states to become self-consistently dependent on the doubles amplitudes as

B7a
$$\epsilon_{a}\delta_{ab}\to \epsilon_{a}\delta_{ab}-\frac{1}{2}\underset{ijc}{\sum }\left\langle ij\left|\right|bc\right\rangle t_{ij}^{ca}=F_{ab}$$

B7b
$$\epsilon_{i}\delta_{ij}\to \epsilon_{i}\delta_{ij}+\frac{1}{2}\underset{kcd}{\sum }\left\langle ik\left|\right|cd\right\rangle t_{jk}^{cd}=F_{ij}$$

B7c
$$\left\langle ij\left|\right|kl\right\rangle \to \left\langle ij\left|\right|kl\right\rangle +\frac{1}{2}\underset{cd}{\sum }\left\langle ij\left|\right|cd\right\rangle t_{kl}^{cd}=\chi_{ij,kl}$$

B7d
$$\left\langle ja\left|\right|bi\right\rangle \to \left\langle ja\left|\right|bi\right\rangle +\underset{kc}{\sum }\left\langle jk\left|\right|bc\right\rangle t_{ik}^{ac}=\chi_{ja,bi}$$

Using these quantities, we write the modified self-consistent interaction matrices as

B8
$$\begin{array}{ll} \left({\mathrm{K̅}}_{ija,klb}^{<,2h1p}+{\mathrm{C̅}}_{ija,klb}^{<,2h1p}\right) & =F_{ik}\delta_{ab}\delta_{jl}+F_{jl}\delta_{ab}\delta_{ik}-F_{ba}\delta_{ik}\delta_{jl}\\ & -F_{il}\delta_{ab}\delta_{jk}-F_{jk}\delta_{ab}\delta_{il}+F_{ba}\delta_{il}\delta_{jk}\\ & +\chi_{ib,al}\delta_{jk}+\chi_{jb,ak}\delta_{il}-\chi_{ij,kl}\delta_{ab}\\ & -\chi_{ib,ak}\delta_{jl}-\chi_{jb,al}\delta_{ik} \end{array}$$

Resolving these equations in the self-energy Riccati equation (eq 48) yields the full CCD amplitude equations.
